# Floristic Account of Apocynaceae (Gentianales) in Tunisia: New Additions to National and North African Flora with Nomenclatural Updates and Taxonomic Notes

**DOI:** 10.3390/plants14030318

**Published:** 2025-01-22

**Authors:** Ridha El Mokni, Duilio Iamonico

**Affiliations:** 1 Laboratory of Botany, Cryptogamy and Plant Biology, Department of Pharmaceutical Sciences ‘A’, Faculty of Pharmacy of Monastir, University of Monastir, Avenue Avicenna, Monastir 5000, Tunisia; ridhaelmokni@yahoo.fr; 2 Laboratory of Forest Ecology, National Research Institute of Rural Engineering, Water and Forests (INRGREF), Institution of Agricultural Research and Higher Education (IRESA), University of Carthage, Ariana 2080, Tunisia; 3 Department of Environmental Biology, University of Rome Sapienza, Piazzale Aldo Moro 5, 00185 Rome, Italy

**Keywords:** *Asclepias*, *Carissa*, *Cascabela*, *Cryptostegia*, chorology, new records, nomenclature, North Africa, typification

## Abstract

A taxonomic revision of the family Apocynaceae (Gentianales) from Tunisia is presented. Field surveys carried out during the last two decades allowed us to identify new records at the national level, i.e., *Asclepias curassavica*, *Carissa macrocarpa*, *Cascabela thevetia*, and *Cryptostegia grandiflora* (the latter three are new to the non-native Mediterranean and North African woody vascular flora). The genus *Asclepias* is new at a national level, whereas *Carissa*, *Cascabela*, and *Cryptostegia* are recorded here for the first time in the Mediterranean basin. Information is provided about nomenclature (accepted names, main synonyms, and types), morphology, chromosome number, chorology, occurrence in Tunisia, habitat, phenology, and taxonomic annotations, and original photos are prepared. Diagnostic keys relating to generic and species ranks are also given. Using typifications, the identity of the Linnaean names *Cynanchum erectum* and *C. monspeliensis*, Pourret’s *Vinca difformis*, and Roxburgh’s *Nerium grandiflorum* is discussed.

## 1. Introduction

Apocynaceae Juss. (Gentianales Juss. ex Bercht. and J.Presl) is one of the largest angiosperm families, comprising about 400 genera and 4500–5100 taxa. These are classified into five subfamilies: Apocynoideae Burnett (77 genera, 860 species), Asclepiadoideae Burnett (214 genera, 2365 species), Periplocoideae Endl. (31 genera, 180 species), Rauvolfioideae Kostel. (79 genera, 859 species), and Secamonoideae Endl. (9 genera, 170 species). The family has a largely tropical to warm temperate distribution [1]. These plants are typically laticiferous, producing various alkaloids and cardenolides (copious milky latex). In addition to many well-known species widely cultivated as ornamentals due to their beautiful flowers, some of these have many medical properties, or also serve as a source of food for butterflies [2].

Morphologically, the Apocynaceae subfamily usually features woody taxa with simple, opposite, or verticillate leaves (rarely alternate in distal part); flowers are mostly actinomorphic, bisexual, and pentamerous, with the calyx being mostly 5-lobed, connate at least at the base, imbricate, or valvate in bud; the corolla is connate at the base, rotated, campanulate, infundibuliform, and sinistrorse (rarely valvate with a corolline and/or gynostegial corona); stamen 5 alternates with the corolla lobe; the gynoecium of 2 apocarpous or hemisyncarpous carpels is superior or rarely subinferior; fruit are variously ornamented, being smooth, muricate, and winged, and are mostly of 2 dehiscent distinct mericarps, each including numerous seeds on the parietal placenta. These are usually seeds with a distal tuft of silky hairs [2,3]. Moreover, plants of this family are known for their high toxicity due to the presence of cardiac glycosides [4,5].

The Tunisian members of Apocynaceae, sensu APG [1], currentlycomprise 10 genera and 12 species (see, e.g., [6,7,8]) belonging to four subfamilies, i.e., Apocynoideae, Asclepiadoideae, Periplocoideae, and Rauvolfioideae. The latter group was also more recently added to the floristic diversity of the country, which was represented only by the tribe Vinceae Duby with both of its subtribes, i.e., Vincinae M.E. Endress (*Vinca* L. spp.) and Catharanthinae Pichon ex Boiteau [*Catharanthus roseus* (L.) G.Don] (see, e.g., [9,10]).

In continuity with the ongoing floristic research on the flora of Tunisia (see, e.g., [10,11,12,13,14,15,16,17,18,19,20,21,22,23]), we found subpopulations of four species belonging to Apocynaceae which are still not recorded for Tunisia.

## 2. Material and Methods

The work is based on field surveys that were carried out by the first author (REM) in Tunisia over the last two decades (period 2004–2024); 34 sites were surveyed, and they were selected mainly based on the land use (artificial surfaces, e.g., railways, hedges of roads, walkways, streams occurring in towns, etc.). Collected specimens were prepared according to standard practice [pressing/drying, identification (also performed on live plants), labeling, and mounting on a standard herbarium sheet of ca. 30 × 45 cm) and deposited at the personal collection of one of the authors (R. El Mokni), in RO (herbarium acronym follows Thiers [24]), in the herbarium of the Faculty of Sciences of Bizerta (not listed in Index Herbariorum) and, from 2015, in the Herbarium of Monastir University (not listed in *Index Herbariorum*) [continuously updated]); conditions for storage include low and constant temperatures (about 16 °C), low humidity (about 35%), and low light. We also carried out an analysis of the pertinent literature (floras, taxonomic works, and protologues of the names investigated, including works cited therein) and an examination of specimens linked to the protologues and the herbarium. We studied original material of species and preserved in the herbaria A, ALF, BM, C, FI, G, GUAD, LY, LINNMPU, NOU, P, REN, and US (acronyms according to [24]). Descriptions are given with details for some taxa that do not figure (or lack amendation) in the flora of Tunisia [6] and, for new national records, those that are not frequently found in the accessible literature. Each entry includes the currently accepted names of subfamilies, tribes, subtribes (when existing), genera, and prioritized species names. If appropriate, these are accompanied by one or more accepted synonyms. This paper enumerates taxa in alphabetical sequence.

A map for all localities from where Tunisian Apocynaceous vouchers were sampled was prepared using coordinates from our GPS-enabled camera and also from Google Earth (https://www.google.it/intl/it/earth/index.html; accessed: 17 January 2025).

The nomenclature of articles cited throughout the text follows the *Shenzhen Code* [25].

## 3. Results and Discussion

A floristic account of the diversity of Tunisian Apocynaceous includes 16 different species, which can be classified into 14 genera, 7 subtribes, 8 tribes and 4 subfamilies; 4 genera are new as Tunisian flora. A key to subfamilies of Apocynaceae in Tunisia is presented hereafter.

1. Anthers adnate to style head; corolla lobe aestivation in bud typically dextrorse (overlapping to the right) or valvate, rarely sinistrorse; fruit always as a pair of follicles; seeds small, compressed, almost always with coma (tuft of hairs) at one end21. Anthers free from style head; corolla lobe aestivation in bud typically sinistrorse (overlapping to the left), rarely dextrorse; fruit as a berry, drupe, follicle, or capsule; seeds naked, with wings, or arils, but almost never with coma at one endRauvolfioideae2. Nectaries, if present, in ring around base of ovary; anthers 4-1ocular; pollen almost always shed as monads; style head secretions for pollen transport normally a foamy adhesive or gummy, undifferentiated translatorsApocynoideae2. Nectaries located in alternistaminal troughs on staminal feet or staminal tube; anthers 2-locular; pollen shed as tetrads or gathered into pollinia; style-head secretions for pollen transport forming differentiated translators with sticky end (viscidium) or consisting of a rigid clip (corpuscle) and two flexible arms (caudicles)33. Anthers 4-1ocular, pollen shed as tetrads or, if gathered into pollinia, then without waxy outer wallPeriplocoideae3. Anthers 2-1ocular, pollen enclosed in pollinia covered by waxy outer wall (ectexine)Asclepiadoideae

**A. Apocynoideae** Burnett, Outlines Bot. 1012, 1095, 1104 (1835).

In Tunisia, this subfamily is only represented by the tribe Nerieae Baill., the subtribe Neriinae Benth. and Hook.f., and the genus *Nerium* L.

**A.1. Nerieae** Baill., Hist. Pl. 10: 166, 198 (1889).

**A.1.1. Neriinae** Benth. and Hook.f., Gen. Pl. 2: 688 (1876).

**A.1.1.1. *Nerium*** L., Sp. Pl. 1: 209 (1753).

***Nerium oleander*** L., Sp. Pl.: 209 (1753).

Lectotype [designated by Stearn in Davis [26] (pag. 159)]. Herb. Clifford: 76, Nerium 1 [beta] (BM000558145!, image available at https://data.nhm.ac.uk/record/d579f8b6-c29e-4f6d-8060-391d2fd907f8/4072/1654074341008; accessed on 17 January 2025).

**Description.** *Nerium oleander* is a small drought-tolerant evergreen tree 2–5 m in height; leaves simple, arranged in whorls of three, blade narrowly elliptic, usually 8–18 cm long, surface finely veined; flowers many, continuously though the year, in dense terminal clusters; corolla of fused petals, funnel-shaped, 3.5–5.5 cm long, with a spreading limb 4–6 cm across, five-lobed, or double-flowered, with a ring of filamentous appendages in the center, pink, purple, or white; fruit pod-like, cylindrical, usually 7–12 cm long, filled with hairy seeds. For more details see, e.g., [27].

**Phenology in Tunisia.** Flowering and fruiting plants have been seen throughout the year for many years.

**Global distribution and occurrence in Tunisia.** *Nerium oleander* is native to the Mediterranean region, including northern Africa, southern Europe, and South Asia, whereas it is considered alien in Africa, Eastern Asia, Mexico, central America, southern Brazil, and southwestern Australia [28,29,30]. In fact, it is widely used as an ornamental plant (screens, hedging highways, beaches, etc.) due to its profuse flowering, which is long-lasting, along with its moderate hardiness [31,32]. In Tunisia, the plant is the most widespread taxon of the family occurring throughout the whole country, and is especially prevalent along roadsides and water courses (the species is to be considered native and in expansion in the country). Along water courses, it becomes sporadically invasive in Kroumiria, Mogods, Cap Bon, and the Tunisian Dorsale (pers. obs. by the first author).

**Specimens examined.** TUNISIA. Jendouba: Ghar Dimaou (El Feidja), 36°29′11″ N, 008°18′28″ E, 690 m a.s.l. within ripicolous habitats, 18.10.2001, *El Mokni s.n.* (Herb. Univ. Bizerta), Tabarka (El Khedheyria), 36°52′16″ N, 008°42′23″ E, 69 m a.s.l. ruderal habitats, *El Mokni s.n.* (Herb. El Mokni, RO); Monastir, Jemmal, 35°37′42″ N, 010°45′39″ E, 25 m a.s.l. ruderal habitats, *El Mokni s.n.* (Herb. El Mokni).

**B. Asclepiadoideae** R. Br. ex Burnett, Outlines Bot.: 1012, 1095, 1103 (1835).

In Tunisia, this subfamily includes 2 tribes (Asclepiadeae and Ceropegieae) and 3 subtribes (Asclepiadinae, Stapeliinae, and Cynanchinae) with 7 genera (*Asclepias*, *Apteranthes*, *Calotropis*, *Cionura*, *Cynanchum*, *Gomphocarpus* and *Pergularia*) and 7 species. A key to genera of the subfamily Asclepiadoideae in Tunisia is presented:

1. Non-thorny cactiform succulents with rudimentary leaves on the young parts
*Apteranthes*
1. Non-succulent, leafy plants22. Leaves heart-shaped at the base, tomentose or glabrous plants32. Leaves not heart-shaped, glabrescent plants43. Plant tomentose with blue-whitish color due to dense short hair; follicles broadly globose to ovoid-acuminate, covered in fleshy bristles
*Pergularia*
3. Plant glabrous; follicles narrowly ovoid, acuminate, glabrous
*Cionura*
4. Slender herbs, sometimes tending to twine at tip; corona cupulate to cylindrical, or if deeply 5-lobed, lobes always joined laterally at base, fleshy, simple except sometimes for adaxial appendage
*Cynanchum*
4. Robust herbs to small trees; corona of 5 free, fleshy, often elaborate lobes55. Leaves ovate to obovate, 7–30 × 3–15 cm, with cordate to amplexicaul base; corona lobes each with an upcurved dorsal spur at base
*Calotropis*
5. Leaves lanceolate, 5–15 × 0.5–4.0 cm, with cuneate to tapering base; corona lobes without spur at base66. Flowers erect; corolla red or purple; corona bright red or yellow; follicles smooth, 1.0–1.5 cm thick
*Asclepias*
6. Flowers pendulous; corolla whitish; corona purplish; follicles with soft spines or bristles, 2.5–5.0 cm thick
*Gomphocarpus*


**B.1. Asclepiadeae** Duby, Bot. Gall. 1: 323 (1828).

**B.1.1. Asclepiadinae** Decne. ex Miq., Fl. Ned. Ind. 2: 484 (1857).

**B.1.1.1. *Asclepias*** L., Sp. Pl.: 214 (1753).

***Asclepias curassavica*** L., Sp. Pl.: 215 (1753) ≡ *Asclepias bicolor* Moench, Methodus: 717 (1794), *nom. superfl. et illeg.* (Art. 52.2 of ICN) ≡ *Asclepias aurantiaca* Salisb. in Prodr. Stirp. Chap. Allerton: 150 (1796), *nom. superfl. et illeg.* (Art. 52.2 of ICN) ≡ *Asclepias nivea* L. var. *curassavica* (L.) Kuntze, Revis. Gen. Pl. 2: 418 (1891).

Lectotype [designated by Woodson [33] (pag. 59)]. Herb. Linn. 310.19 LINN! image available at https://linnean-online.org/3365/, accessed on 17 January 2025).

**Figure 1:**
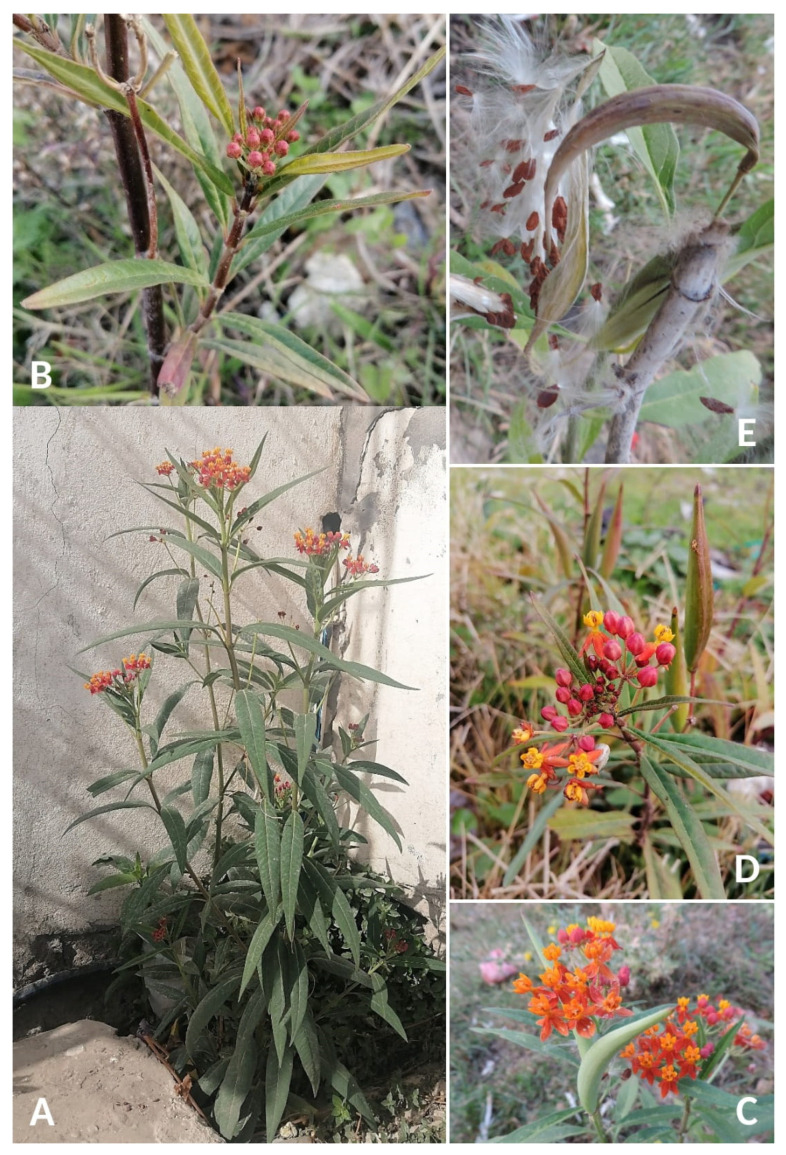
*Asclepias curassavica* in Tunisia. (**A**) Habit; (**B**) inflorescence before blooming; (**C**,**D**) inflorescences in bloom with indehiscent fruit; (**E**) dehiscent follicle showing seeds bearing long coma (photographs by R. El Mokni).

**Description** (Figure 1). *Asclepias curassavica* is a herbaceous annual or short-lived perennial plant (chamaephyte, subshrub) with stout stems up to 120 cm high, frequently rather woody toward the base, simple or branched, minutely arachnoid–tomentulose when young, soon glabrate; leaves opposite, petiolate, elliptic-lanceolate, acute to acuminate, the base acute to obtuse, 5–12 cm. long, 1–3 cm broad, minutely pilosulose when very young, soon glabrate, thinly membranaceous; petioles 0.5–1.0 cm long. Inflorescences solitary at the upper nodes, several- to many-flowered; peduncles 3–6 cm long; pedicels 1–2 cm long; flowers rather large and showy; calyx lobes narrowly lanceolate, 2–3 mm long; corolla reflexed-rotate, bright crimson, rarely yellow or white, the lobes 5–10 mm long; gynostegium long-stipitate, deep yellow, the column cylindric or conic, 2–3 mm long and 1 mm broad at the base, the hoods cucullate, distinctly stipitate, broadly oblong, rounded at the tip, 3–5 mm long, the horn basal, narrowly acicular, 4–5 mm long, slightly incurved over the anther head; anther head cylindrical, 2–3 mm long, 1.5–2.5 mm broad; follicles erect on erect pedicels, narrowly fusiform, 6–10 cm long, smooth, glabrous; seeds broadly oval, 5–7 mm long, the white coma about 2–3 cm long. More details are available, e.g., in [33,34,35,36].

**Phenology in Tunisia.** Flowering and fruiting plants have been seen from July to February for many years.

**Iconography.** Shamim et al. ([34] 2010, Figure. 1: 71)

**Chromosome number.** 2n = 2x = 22 [37,38].

**Global distribution and occurrence in Tunisia.** *Asclepias curassavica* has a native distribution range from Mexico to tropical South America, whereas it is considered as an exotic species in the other continents ([30] POWO 2024). The plant was introduced in many countries and is widely naturalized in tropical Asia, Africa, and Australia [30,39,40,41]. In North African, the species is only reported in the Canary Islands and Morocco [39].

*Asclepias curassavica* was reported here for the first time from in Tunisia in the following four localities:

> This was first recorded in the wild in the northeastern part of Tunisia (Bizerta region, Cité Sahha, 19.12.2020), with about ten flowering–fruiting individuals found within an abandoned field of about one hectare.
> The second record of this in the wild was made in the northwestern part of Tunisia (Bizerta region, Sidi Salem, 16.02.2021), where about 5 blooming–fruiting individuals were found within waste places in an area of about 200 m^2^.
> The third record of this in the wild was made in the northwestern part of Tunisia (Tabarka region, Larmèel, 05.07.2022), where a dozen of flowering–fruiting individuals were found along the roadside to the right along the road leading to the sea within an area of about 100 m^2^.
> The fourth locality was also in the northwestern part of the country (Tabarka region, Jaballah, 13.07.2023), where we detected several flowering individuals of almost 50–80 cm high within ruderal habitats in an area of about 100 m^2^.

Although subpopulations of *Asclepias curassavica* are currently sporadic and not very widespread in Tunisia (the species can be considered as casual in the country), their further spread and naturalization are likely. Therefore, early detection and rapid response measures should be taken, when deemed necessary, to ensure that they will not become naturalized or even invasive.

**Taxonomic notes.** *Asclepias curassavica* differs from other closely related species due to its flowers with a bright red corolla and orange or yellow corona, with a prominent tooth arising from the cavity and arching over the stylar head. Related species have white, yellow, green, or brownish corollas with variously colored corona, and are missing corona tooth, which are sometimes inconspicuous and included within the cavity of the lobe. It is an annual or short-lived perennial with fibrous, non-tuberous rootstock [42].

**Specimens examined.** TUNISIA. Bizerta: Bizerta-city (Cité Sahha), 37°17′30″ N, 009°51′55″ E, 7 m a.s.l. ruderal habitats, 19.12.2020, *El Mokni s.n.* (Herb. El Mokni!), ibidem, Sidi Salem, 16.02.2021, *El Mokni s.n.* (Herb. El Mokni!); Jendouba, Tabarka, Larmèel, 36°57′02″ N, 008°42′41″ E, 69 m a.s.l. ruderal habitats, 05.08.2022, *El Mokni s.n.* (Herb. El Mokni!), Jaballah, 36°56′54″ N, 008°45′34″ E, 2 m a.s.l. ruderal habitats, 13.07.2023, *El Mokni s.n.* (Herb. El Mokni, RO).

**B.1.1.2. *Calotropis*** R. Br., Asclepiadeae: 28 (1810).

***Calotropis procera*** (Aiton) W.T.Aiton, Hortus Kew. 2: 78 (1811).

Lectotype [designated by Ali [43] (pag. 287–290)]. [Icon] *Asclepias gigantea* in Jacquin (1768: t. 69 image available at https://bibdigital.rjb.csic.es/viewer/12016/?offset=#page=47&viewer=picture&o=bookmark&n=0&q=; accessed on 17 January 2025).

**Description.** *Calotropis procera* is a drought-resistant, salt-tolerant to a relatively high degree, shrub or small tree ca. 2.5 m (up to 6 m) high; stem usually simple, rarely branched, woody at base and covered with a fissured, corky bark; branches somewhat succulent and densely white tomentose, early glabrescent (all parts of the plant exude a white latex when cut or broken); leaves opposite, simple, subsessile, stipule absent; blade oblong–obovate to broadly obovate, 5–30 × 2.5–15.5 cm, apex abruptly and shortly acuminate to apiculate, base cordate, margins entire, succulent, white tomentose when young, later glabrescent and glaucous; inflorescence a dense, multiflowered, umbellate cyme arising from the nodes and appearing axillary of the uppermost leaves or terminal; flowers hermaphroditic, pentamerous, pedicle 1–3 cm long; calyx 5-lobed, shortly united at the base, lobes ovate, 4–7 × 3–4 mm, glabrescent; corolla five-petalled, white with purple tips inside and have a central purplish crown; fruit, a simple, fleshy, inflated, subglobose to obliquely ovoid follicle up to 10 cm or more in diameter; seeds numerous, flat obovate, 6 × 5 mm, with silky white pappus 3 cm or more long, these can be carried long distances by the wind.

**Phenology in Tunisia.** Flowering and fruiting plants have been seen from October to December.

**Iconography.** [44] (Figure. 104, A–D: 212).

**Global distribution and occurrence in Tunisia.** *Calotropis procera* is native to north to tropical southwestern Asia, and Macaronesia, while it is an alien in the Americas, south-eastern China, and Australia [30,45]. In these parts of the world, it is considered as a noxious weed and harmful to natural biodiversity and its invasiveness in Brazil is well documented (see, e.g., [46,47]). It has increasing popularity as a medicinal plant, which is likely to lead to further spread. In inland North Africa, the plant is reported as native from Morocco to Egypt [48], except for Tunisia, where the species was officially reported for the first time in 2006 ([7] Meddour and El Mokni) from the governorate of Medenine (southeast of the country). The species seemed to spread rapidly and extensively due to wind and animal activities in the south of Tunisia; many observations were made by the first author in the governorates of Medenine and Tatouine (southern part of the country). The species can be considered as naturalized in the country.

**Specimens examined.** TUNISIA. Medenine: Gordhab, 33°05′37″ N, 010°31′08″ E, 154 m a.s.l., sandy soils, 20.03.2014, *El Mokni s.n.* (Herb. Univ. Monastir, RO); Around Ben Guerdane, 33°04′05″ N, 011°15′15″ E, 15 m a.s.l., sandy soils, 03.01.2025, *El Mokni s.n.* (Herb. Univ. Monastir); Tataouine, around El Borma, 31°46′14″ N, 009°22′44″ E, 263 m a.s.l., sandy soils, 15.05.2021, *El Mokni s.c.* (Herb. Univ. Monastir).

**B.1.1.3. *Gomphocarpus*** R. Br., Asclepiadeae: 26 (1810).

***Gomphocarpus fruticosus*** (L.) W.T.Aiton, Hortus Kew. 2: 80 (1811) ≡ *Asclepias fruticosa* L., Sp. Pl., ed. 1: 216 (1753) ≡ *Gomphocarpus fruticosus* (L.) R.Br., Mem. Wern. Nat. Hist. Soc. 1: 38 (1810).

Lectotype [designated by Woodson [33] (pag. 151). Herb. Linn. 310.33 (LINN! image available at https://linnean-online.org/2588/#?s=0&cv=0; accessed on 17 January 2025).

**Description.** *Gomphocarpus fruticosus* is a shrub up to 2 m tall; stems are puberulent; leaves opposite, short petiolate; leaf blade linear or linear-lanceolate, 6–10 cm × 5–8 mm, glabrous, apex acuminate, margin revolute; lateral veins inconspicuous; inflorescences extra-axillary with 4–7(–12) flowers in nodding umbel; flower buds globose; sepals 2–5 × 0.6–1.3 mm, lanceolate or triangular, puberulent; corolla white, reflexed, glabrous outside with lobes 5–8 × 3–5 mm, broadly ovate or elliptic, minutely papillate and ciliate along margins; corona lobes dark, hoodlike; follicles 4–7 × 1.5–2.5 cm, inflated, ovoid, pubescent apex acuminate, long beaked, spines of pericarp soft, ca. 1 cm long; seeds ovate; coma ca. 3 cm long.

**Phenology in Tunisia.** Flowering and fruiting plants have been seen from May to November/December for many years.

**Global distribution and occurrence in Tunisia.** *Gomphocarpus fruticosus* is native to southern and eastern Africa and the Arabian Peninsula, whereas it is exotic in the eastern U.S.A., south America, Mediterranean area, central and south Asia, and east Australia [30]. In Tunisia, the species was historically reported as being subspontaneous in the Tunisian ridge (Zaghouan governorate) and within the southeastern of the country (Gabès governorate) and no new reports are recorded. The species is to be considered native but rare in the country.

**Notes.** *Gomphocarpus fruticosus* shows high morphological similarities with *G. physocarpus*. The two species can mainly be distinguished by the following differences: the former has narrowly egg-shaped (i.e., ovoid) and slightly curved (i.e., falcate) fruit that gradually taper to a short, curved beak, whereas the latter has rounded (i.e., globose or sub-globose) fruit that come to an abrupt point and often have a sunken (i.e., indented) tip with a tiny beak [49] (pag. 781).

**Specimens examined.** TUNISIA. Gabès: Gabès surroundings (Oued sourrague), 33°49′36″ N, 010°08′52″ E, 5 m a.s.l., sandy soils, 11.10.2003, *El Mokni s.n.* (Herb. Univ. Bizerta, RO).

**B.1.1.4. *Pergularia*** L., Syst. Nat., ed. 12, 2: 135, 191 and Mant. Pl.: 8, 53 (1767).

***Pergularia tomentosa*** L., in Mant. Pl.: 53 (1767).

Lectotype (designated by Goyder [50] (pag. 248). Yemen, Taizz, Forsskål 972 (C10001710! image available at https://plants.jstor.org/stable/viewer/10.5555/al.ap.specimen.c10001710?loggedin=true; accessed on 17 January 2025).

**Description.** *Pergularia tomentosa* is a perennial herb that climbs to become semi-erect. The whole plant is blue-whitish due to dense short hair. A detailed description was given by Goyder [50] (pag. 248).

**Phenology in Tunisia.** Flowering and fruiting specimens have been seen throughout the year for many years.

**Global distribution and occurrence in Tunisia.** *Pergularia tomentosa* is widely distributed across the Sahara Desert and western Asia, al the way to India, but it has never been found as alien being outside of its native range [30,50]. In Tunisia, the species has a very widely distribution from the southern desert to all central governorates, with high abundance throughout the year, and it is considered native but is subject to expansion in the country.

**Specimens examined.** TUNISIA. Monastir: Zeramdine, 35°33′34″ N, 010°44′01″ E, 123 m a.s.l., ruderal, 19.10.2016, El Mokni s.n. (Herb. El Mokni!), Ksibet El Mediouni, 35°40′46″ N, 010°50′39″ E, 35 m a.s.l., along railways, 19.10.2023, *El Mokni s.n.* (Herb. El Mokni, RO); Kairouan, Cherarda, 35°08′54″ N, 010°02′22″ E, 105 m a.s.l., ruderal, 24.12.2024, *El Mokni s.n.* (Herb. El Mokni!); Gafsa, Sened, 34°27′44″ N, 009°15′44″ E, 605 m a.s.l., ruderal, 25.12.2024, *El Mokni s.n.* (Herb. El Mokni).

**B.1.2. Cynanchinae** K. Schum. in Engl. and Prantl, Nat. Pflanzenfam. 4(2): 209, 245 (1895).

**B.1.2.1. *Cynanchum*** L., Sp. Pl.: 212 (1753).

***Cynanchum acutum*** L., Sp. Pl.: 212 (1753) subsp. ***acutum***

Lectotype (designated by Ali in Nasir and Ali [51] (pag. 12). Herb. Linn. 308.3 (LINN! image available at https://linnean-online.org/2571/; accessed on 17 January 2025).

= *Cynanchum monspeliacum* L., Sp. Pl.: 212 (1753).

Lectotype **(designated here)**. Herb. Linn no. 308.6 (LINN! image available at https://linnean-online.org/2959/#?s=0&cv=0; accessed on 17 January 2025).

**Description.** *Cynanchum acutum* is a climbing subshrub or shrub; stems many-branched and woody at base, twining to 3 m, villous to puberulent, sometimes glabrous, petiole 0.5–4.0 cm; leaf blade hastate, hastate-cordate, or oblong-hastate, 1.3–6.0(–15) × 1.1–4.5(–8) cm, papery, base auriculate, apex acute to long acuminate, basal lobes recurved, parallel or divergent, ciliate, glabrous or densely pubescent along veins; basal veins 5–7, lateral veins ca. 3 pairs; inflorescences racemelike; peduncle 0.5–5.0 cm, rachis to 7 cm, pedicel 4–8 mm; sepals ovate, 1.5–2.0 × 0.5–0.9 mm, puberulent outside, glandular inside; corolla white outside, white to purple inside; tube ca. 1 mm; lobes narrowly ovate or oblong, ca. 4.0 × 1.3–2.0 mm, obtuse, glabrous; corona cylindric, margin 5-lobed; lobes 3-fid with middle segment long threadlike, interior with 5 short appendages included within tube, anthers nearly square, appendages ovate, pollinia oblong, stigma head subapiculate; follicles lanceolate to linear, 6.5–13.0 cm × 8–10 mm, puberulent; seeds oblong-ovate, 5.0–7.5 × 2.0–2.5 mm; coma 2–3 cm long.

**Phenology in Tunisia.** Flowering and fruiting plants have been seen from June to November/December for many years.

**Global distribution and occurrence in Tunisia.** The native range of this species is a wider area from the Mediterranean basin to Siberia and north China plus the Canary Islands (Lanzarote); it has never been recorded as an alien species out of its native rage [29]. In Tunisia, the species is reported from the extreme southwestern (Tozeur governorate) to the northeastern part of the country (Cap Bon, Nabeul governorate), except the extreme northern part and Kroumiria. The last recent reports were recorded in 2024 from Gabès governorate (southeastern part). The species is to be considered native and rare in the country.

**Typification of the name *Cynanchum monspeliacum*.** Linnaeus [52] (pag 212) published *Cynanchum monspeliacum* by providing a short diagnosis (“CYNANCHUM caule volubili herbaceo, foliis reniformis-cordatis acutis”) taken directly from Linnaeus [52] (pag. 79), Royen [53] (pag. 409), and Sauvage [54] (pag. 133). Synonyms were cited from Bauhin [55] (pag. 294, “Scammonea monspeliaca, foliis rotundioribus”) and Clusius [56] (pag. 126, “Apocynum IV [as “IIII” in Clusius’s Rariorum plantarum historia] latifolium: Scammonea valentina”). The provenance (“Habitat in Hispania & Narbonae maritimis“) was also given, followed by the symbol of Jupiter (♃), which means that the plant is perennial. Clusius [56] (pag. 126) provided an illustration (image available at https://bibdigital.rjb.csic.es/viewer/13800/?offset=#page=139&viewer=picture&o=bookmark&n=0&q=, accessed on 21 July 2024), which is original material for *Cynanchum monspeliacum*.

We traced two specimens, one at BM (barcode BM000558171; image available at https://raw.githubusercontent.com/NaturalHistoryMuseum/CliffordHerbarium-imgs/master/BM000558171.JPG, accessed on 22 July 2024) and one at LINN (no. 308.6). These specimens were each plants with leaves and flowers (few developed in BM000558171) and, respectively, bore the annotations “Periploca monspeliaca foliis rotundioribus T. 93” [where “T.” refers to Tournefort [57] (pag. 93) cited by Linnaeus [52] (pag. 79) in Hortus Cliffortianus] and “4 monspeliacum” (bottom-center of the sheet). BM and LINN specimens are ante-1753 additions to the collection and, therefore, part of the original material [58]. Both the exsiccata and Clusius’ illustration of Apocynum IV latifolium morphologically matches the Linnaean diagnosis concerning the habit (a scandent herb) and the base of the leaf blades (cordate), whereas the apex of the leaves is a gradually diminishing point or apex (BM specimen) or short to long acuminate (Clusius’s illustration and LINN specimen). However, it is not acute, as stated in the protologue. It should be noted that the character “foliis acuminatiis” was reported by Linnaeus [52] (pag. 212) as diagnostic for *C. suberosum* L., but its lectotype (Dillenius’ illustration no. 229 in Hortus Elthamensis (see https://bibdigital.rjb.csic.es/viewer/11970/?offset=#page=169&viewer=picture&o=bookmark&n=0&q=, accessed on 23 July 2024)) does not show leaves with strictly acuminate apexes in the modern sense (i.e., tapered into a slender and long tip) and they appear to be a gradually diminishing point or apex (as with the BM specimen of *C. monspeliacum*). All things considered, Linnaeus’ concepts of the terms “acute” and “acuminate” seem to overlap partially and we do not consider the “foliis … acutis” of *C. monspeliansis* in contrast with the shape of the leaves in BM and LINN specimens. Among the elements found (Clusius’ illustration and BM and LINN specimens), we here designate LINN-308.6 as the lectotype of the name *Cynanchum monspeliacum*, since it includes well-developed flowers.

Based on the literature, *Cynanchum monspeliacum* is often considered as a heterotypic synonym of *Cynanchum acutum* subsp. *acutum* (see, e.g., [30,49,59]). It is important to note that these two names were both published both in volume no. 1 of the 1st edition of *Species Plantarum* [51] (pag. 212). Based on the diagnoses given for *C. acutum* and *C. monspeliacum*, no clear differences can be found ([52] (pag.: 212); respectively, “CYNANCHUM caule volubili herbaceo, foliis cordato-oblongis glabris” and “CYNANCHUM caule volubili herbaceo, foliis reniformis-cordatis acutis”). By examining the lectotypes of these two names (specimens LINN nos. 308.3 and 308.6, respectively), they appear to be different in terms of the shape of the leaves (cordate with apex acute, which is about two times longer than wide in *C. acutum*, vs. cordate with apex acuminate, which is about as long as wide in *C. monspeliacum*), whereas the structure of the inflorescence is the same in both specimens (raceme-like). Currently, two subspecies are recognized under *Cynanchum acutum*, i.e., subsp. *acutum* and subsp. *sibiricum* (Willd.) Rech.f, which differ from each other by the shape of the leaves, i.e., these are, respectively, broadly ovate–cordate and 1–2 times as long as broad with basal lobes scarcely divergent, vs. hastate–cordate and usually 3 or more times as long as broad, with basal lobes distinctly divergent [26,60]. Both the lectotype of *C. monspeliensis* (LINN-308.36) and the other original elements (Clusius’ illustration and BM000558171) display leaves that are broadly ovate–cordate with ratios of length/width of about 1, whereas the lectotype of *C. acutum* (LINN-308.6) shows leaves that are about 2 times as long as they are broad. Basal lobes are more or less divergent. All things considered, we here confirm that *C. monspeliensis* can be considered as a heterotypic synonym of *C. acutum* subsp. *acutum*.

**Specimens examined.** TUNISIA. Gabès: Gabès-city, 33°53′39″ N, 010°05′56″ E, 8 m a.s.l., ruderal on the hedges of oases, 15.05.2021, *El Mokni s.n.* (Herb. El Mokni, RO).

**B.2. Ceropegieae** Decne. ex Orb., Dict. Univ. Hist. Nat. 3: 339 (1843).

**B.2.1. Stapeliinae** G. Don, Gen. Hist. 4: 106, 109 (1837–1838).

**B.1.2.1. *Apteranthes*** J.C.Mikan, Nova Acta Phys.-Med. Acad. Caes. Leop.-Carol. Nat. Cur. 17: 593 (1835).

***Apteranthes europaea*** (Guss.) Murb., in Acta Univ. Lund. 34(7): 2 (1898) ≡ *Boucerosia europaea* (Guss.) G.Nicholson, Ill. Dict. Gard. 1(5): 205 (1884) ≡ *Caralluma europaea* (Guss.) N. E. Br. in Gard. Chron., ser. 3, 12: 369 (1892) ≡ *Stapelia europaea* Guss., Suppl. Fl. Sicul. Prodr.: 65 (1832) = *Apteranthes europaea* (Guss.) Plowes, Haseltonia 3: 59 (1995), isonym (Art. 6 Note 2 of the ICN).

Lectotype [(designated by Gilbert [61] (pag. 16)]. Italy, Lampedusa, s.d., Gussone s.n. (NAP).

**Description.** *Apteranthes europaea* is a low, succulent subshrub with long stolons running underground. A detailed description was provided recently in [12] (pag. 67) for sub *Ceropegia europaea* (Guss.) Bruyns.

**Phenology in Tunisia.** Flowering and fruiting plants have been seen from March to October(-November) for many years.

**Global distribution and occurrence in Tunisia.** The species has a native range, spanning from the south Mediterranean area of southeastern Spain to North Africa and west Jordan plus Sicily (Linosa and Lampedusa islands) [30]. In Tunisia, the taxon sub *Ceropegia europaea* (Guss.) Bruyns was more recently put under a national review [12] (pag. 68). The species is to be considered native but is subject to rarefaction in the country.

**Specimens examined.** TUNISIA. Kasserine: Sbeitla, 035°14′18″ N, 008°59′11″ E, 638 m a.s.l., low steppes with sandy loam soil, 17.03.2001, *El Mokni s.n.* (herb. Univ. Bizerta); Nabeul: Ben Arous, Dj. Ressas, 36°36′23″ N, 010°20′21″ E, 624 m a.s.l., cracks in limestone/calcareous rocks of scrublands, 30.03.2014, *El Mokni s.n.* (Herb. Univ. Monastir); Sousse: Enfidha, Takrouna, 036°09′09″ N, 010°20′18″ E, 61 m a.s.l., cracks in limestone/calcareous rocks of scrublands, 28.10.2018, *El Mokni s.n.* (Herb. El Mokni!), ibidem, 27.10.2019, *El Mokni s.n.* (Herb. El Mokni!); Kairouan: Aîn Cherichera, 035°37′30″ N, 009°49′42″ E, 209 m a.s.l., cracks in limestone/calcareous rocks within chamaephytes, 21.08.2019, *El Mokni s.n.* (Herb. El Mokni, RO). 

**B.3. Marsdenieae** Benth., Fl. Austral. 4: 325, 333 (1868) emend. Liede-Schumann et al. (2022).

**B.3.1. *Cionura*** Griseb., Spic. Fl. Rumel. 2: 69 (1844). 

***Cionura erecta*** (L.) Griseb., Spic. Fl. Rumel. 2: 69 (1844) ≡ *Cynanchum erectum* L., Sp. Pl.: 213 (1753) ≡ *Marsdenia erecta* (L.) R.Br. in Mem. Wern. Nat. Hist. Soc. 1: 31 (1811) ≡ *Pergularia erecta* (L.) Spreng. in Syst. Veg., ed. 16. 1: 844 (1824). 

Lectotype [designated by Browicz [62] (pag. 9), 10 as “holotype”, **here corrected to lectotype** according to the Art. 9.10 of ICN)]: Herb. Linn. No. 308.12 (LINN! image available at https://linnean-online.org/2965; accessed on 17 January 2025).

**Description.** *Cionura erecta* is a perennial herb or subshrub; stems numerous, to 100 cm tall, often sprawling and forming large mats, white puberulent when young, glabrous later; leaves opposite, long petiolate, green and ± glaucous, fetid; lamina c. 7 × 5 cm, ovate to broadly ovate, usually cordate at base, acute or acuminate at apex; petiole to 5 cm long; inflorescence an axillary or terminal cyme with numerous flowers; peduncle subequal to petiole; calyx small; corolla rotate, white to cream, rarely yellow, glabrous, c. 1 cm in diam., lobes oblong, obtuse, patent at anthesis, slightly overlapping to right in bud; corona segments free, erect, triangular, membranous, reaching height of anthers and opposite to them; gynostegium exposed at base of corolla; follicles c. 8.0 × 1.5 cm, glabrous, narrowly ovoid, acuminate, usually solitary by abortion, rarely both carpels developing; seeds ovate–oblong, with a marginal wing; coma long, silky, white [62,63].

**Phenology in Tunisia.** Flowering and fruiting plants were seen at the end of March 2008. 

**Global distribution and occurrence in Tunisia.** The species has a native range within Europe and Asia, running from southeastern Europe in the Balkan Peninsula to southwestern Asia, and reaching southwestern Iran in the east [30,64,65]. In terms of appearing outside of its native range, within the African continent, the plant was only reported as being introduced in Tunisia [30]. It has been seen as adventitious (casual alien) within the oases of Chenini in Gabès (southeastern part) since 2008 [66] (pag. 412–413). For the moment, the species can be considered as casual in the country. More prospections are needed to clarify its naturalization status.

**Figure 2:**
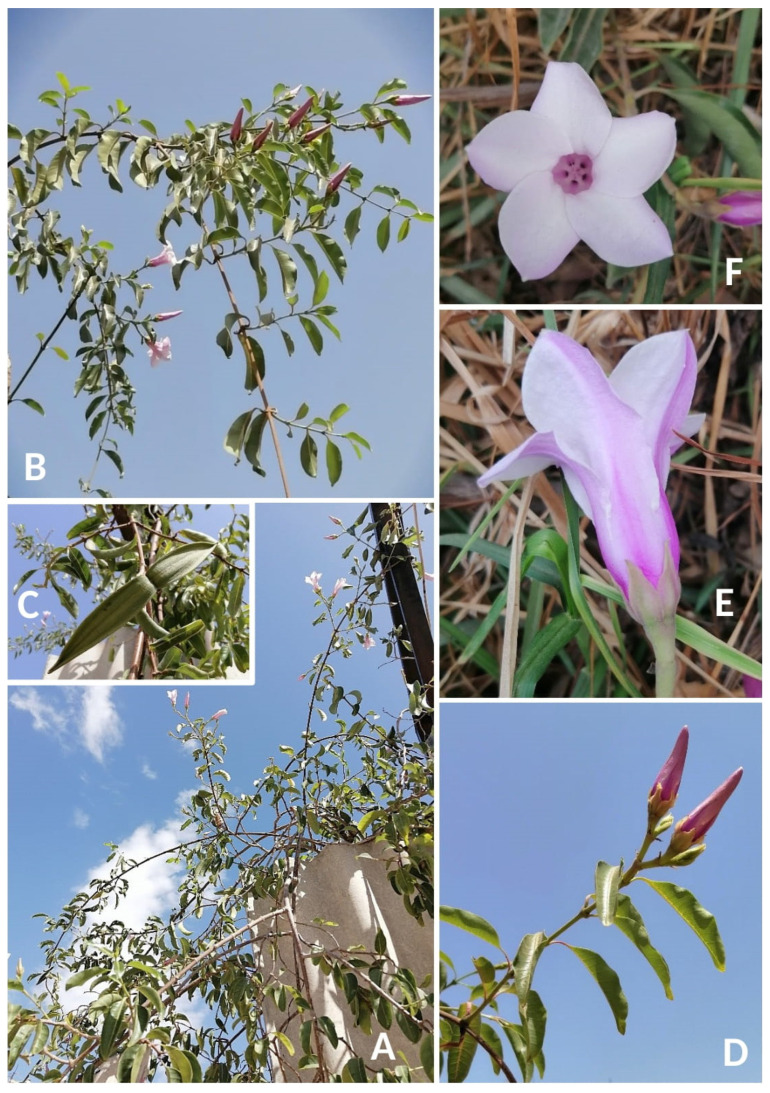
*Cryptostegia grandiflora* in Tunisia. (**A**) Habit; (**B**) flowering climbing stems; (**C**) mature fruit; (**D**) lateral inflorescence with non-blooming flowers; (**E**) side view of a flower; (**F**) a face view of corolla. Photographs by R. El Mokni.

**Notes on the typification of the name *Cynanchum erectum*.** Browicz [62] recognized the genus *Cionura* Griseb. as monotypic, with the single species *Ci. erecta* (L.) Griseb.; he also reported “Type: Herbarium of Linnaeus [LINN.] (Figure 2).”, where figure no. 2 (the caption is “Holotype of *Cionura erecta* from the Herbarium of Linnaeus”) represents the specimen LINN no. 308.12. By checking the protologue of *Ci. erectum* [52] (pag. 213), we can confirm that LINN-308.12 is part of the original material for the name since it bears the original annotation “5 erectum” (bottom center of the sheet), which means that this specimen is an ante-1753 addition to the collection [58]. According to Art. 9.10 of ICN, the use of the term “holotype” by Browicz [62] to denote the type is to be treated as an error to be corrected to “lectotype”.

**Specimens examined.** TUNISIA. Gabès: Chenini, 29.03.2008 (Société botanique de France!), *El Mokni s.n.* (Herb. El Mokni, RO); *ibidem*, 33°51′55″ N, 010°03′07″ E, 30 m a.s.l., climbing on hedges within oases on sandy soils, 29.12.2024, *El Mokni s.n.* (Herb. El Mokni, RO). 

**C. Periplocoideae** Endl., Gen. Pl.: 587 (1838).

In Tunisia, this subfamily only has 1 tribe (Cryptolepideae) with 2 genera (*Cryptostegia* and *Periploca*) and 2 species. A key to the genera of the subfamily Periplocoideae in Tunisia is presented:

1. Flowers in axillary, pseudumbellate clusters; corolla large and showy, funnel-shaped, pink to purple-red; fruit three-keeled, horizontal paired follicles
*Cryptostegia*
1. Inflorescence terminal, few- to many-flowered; corolla tube saucer-shaped, lobes linear, violet or brownish violet on the inside with exserted gynostegia, sometimes with dark-colored glandular center and hairy white spots; fruits tapered from the base with an acute apex, paired follicles horn-like, horizontally divergent
*Periploca*


**C.1. Cryptolepideae** Venter, Taxon 46: 713 (1997).

**C.1.1. *Cryptostegia*** R. Br., Bot. Reg. 5: t. 435 (1820).

***Cryptostegia grandiflora*** Roxb. ex R.Br., Bot. Reg. 5: t. 435 (1819).

Lectotype [designated by Marohasy and Forster [67] (pag. 574)]: t. 435 in Brown 1819 (image available at https://www.biodiversitylibrary.org/page/62012836#page/388/mode/1up, accessed on 24 July 2024).

≡ *Nerium grandiflorum* Roxb., Fl. Ind., ed. 2, 2: 10 (1832)

Neotype **(designated here)**. t. 435 in Brown 1819 (image available at https://www.biodiversitylibrary.org/page/62012836#page/388/mode/1up, accessed on 25 July 2024).

**Description** (Figure 2). *Cryptostegia grandiflora* is a woody twining liana to shrub with scrambling branches, 2–3(–10) m long with abundant white latex in all parts of the plant; branches glabrous, usually with numerous small lenticels; leaf blade elliptic to ovate, 6–9 × 3–5 cm, cuneate to tapering at base, usually acuminate at apex, glabrous; petiole 5–15 mm long, glabrous to rarely slightly hairy; internodes of cymes 5–15 mm long; pedicels 3–7 mm long, usually hairy; bracts 2–7 mm long; calyx lobes narrowly ovate to ovate, 14–20 × 4.2–8.8 mm, with reflexed margins, corolla tube 18–30 mm long; lobes 32–56 × 15–30 mm; corona lobes 8–11 mm high, bifid near apex only or cleft almost to the base; staminal cone 3–4.5 mm high; anthers 2.7–4.5 mm; style 1.3–3.4 mm long; style including style head 4.8–6.8 mm long; follicles 8–13.5 × 2–3.5 cm, glabrous; seeds 5–8 mm long; hairs 3–4 cm long. More detailed morphological description is provided by Klackenberg [68] (pag. 213).

**Phenology.** In Tunisia, flowering and fruiting have been seen from July to November for 4 years. In Madagascar, flowering specimens have been seen during August, October, and November and also from January to March [68] (pag. 215).

**Figure 3:**
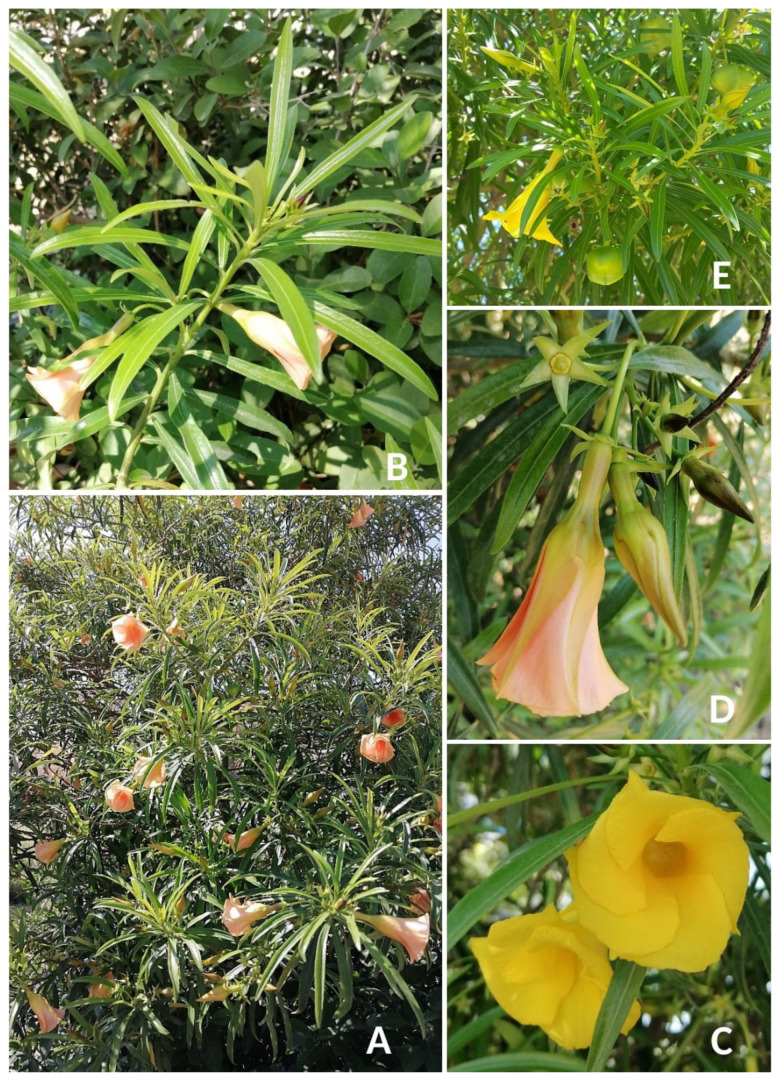
*Cascabela thevetia* in Tunisia. (**A**) Habit; (**B**) flowering lateral stem; (**C**) face view of yellow corolla; (**D**) side view of blooming orange flower with calyx; (**E**) mature capsules in lateral ramification with yellow flowers (photographs by R. El Mokni).

**Iconography.** Klackenberg [68] (Figure 3, pag. 214).

**Global distribution and occurrence in Tunisia.** *Cryptostegia grandiflora* is native to southwest Madagascar, where it mainly occurs as a riverine plant, especially as a climber in the upper story of gallery forests, and around waterholes and at the edge of coastal salt marshes [67]. It was introduced in north Queensland, where it is now a common and highly invasive component of dry rain forest as well as flood plains, from where it invades grazing land. It is mainly present in tropical to subtropical areas where annual rainfall exceeds 400 mm [69]. In Africa, it was reported that the plant was introduced in several countries (including Morocco and Egypt), was naturalized in Botswana, Tanzania, and Zambia, and was only invasive in Ethiopia and South Africa [70].

*Cryptostegia grandiflora* was reported here for the first time in Tunisia from only one locality, the central eastern part of Tunisia (Monastir towards Sousse). There were only two flowering–fruiting climbing individuals on the edges of abandoned old buildings within an area of about 100 m^2^. The species can be considered casual in the country.

**Taxonomic notes.** Recognized from the other known species (*C. madagascariensis* Bojer ex Decne) by its calyx lobes > 13 mm long (vs. calyx lobes ≤ 13 mm long); corona lobes bifid (vs. corona lobes entire); spathe of translator orbicular, obtuse at apex (vs. spathe of translator ovate, acute at apex); leaves always glabrous (vs. leaves sometimes hairy); follicles often more than 10 cm long (vs. follicles shorter than 10 cm) [68]. 

**Typification of the name *Nerium grandiflorum*.** *Nerium grandiflorum* was published twice by Roxburgh [71] (pag. 19) and [72] (pag. 10), but in *Hortus Bengalensis* the name was nudum and, therefore, it was not published validly according to Art. 38.1 of ICN. On the contrary, the presentation of Nerium grandiflorum in Roxburgh’ *Flora Indica* is valid, being described in detail (its provenance was reported as “A native of the Peninsula of India”). In POWO [29], Roxbugh’s taxon was given as “*Nerium grandiflorum* (Roxb. ex R.Br.) Roxb. First published in Fl. Ind., ed. 1832. 2: 10 (1832)”. However, Roxburgh (pag. 10) only reported “*N*. [*Nerium*] *grandiflorum*, R. [Roxburgh]” and, therefore, POWO’s name is not correctly cited. Brown’s *Nerium grandiflorum* appeared in the 5th volume of *Botanical Register* [73] (t. 435), where the author reported the following: “CRYPTOSTEGIA grandiflora … Brown MSS [manuscript]” followed by “*Nerium grandiflorum*. Roxburgh Flor. ind. ined.” In fact, Roxburgh’s *Flora Indica* was published later than Brown’s work, i.e., 1832 vs. 1819). As a consequence, the correct name for this taxon is *Cryptostegia grandiflora* Roxb. ex Br., as correctly reported in POWO [29]. The name “*Nerium grandiflorum* Roxb.”, cited by Brown [73] in synonymy, is invalid according to Art. 36.1b of ICN. It should also be note also that Brown [73] stated “The following is Dr. Roxburgh’s description of the plant”, followed by a description which mostly corresponds to Roxburgh’s one given in *Flora Indica* [72]. Although Brown’s and Roxburgh’s names clearly refer to the same taxon, they are heterotypics and each need the designation of a type. Marohasy and Forster ([67] 1991) proposed that Brown’s illustration no. 435 is the lectotype for *Cryptostegia grandiflora* Roxb. ex Br. and we accept this designation. *Nerium grandiflorum* Roxb. does not appear to be typified. We were not able to trace the original material; therefore, we propose to neotypify the name using the lectotype of Brown’s binomial, thus making the two names homotypics. This procedure is useful when, in addition to a lack of original material, synonymy between two names is a certainty (see, e.g., [74]), as in our case.

**Specimens examined.** TUNISIA. Monastir-Sousse, 35°45′36″ N, 010°44′35″ E, 2 m a.s.l. ruderal habitats, 19-30.10.2020, *El Mokni s.n.* (Herb. El Mokni!), ibidem, 26.07.2021, *El Mokni s.n.* (Herb. El Mokni!), ibidem, 05.10.2021, *El Mokni s.n.* (Herb. El Mokni!), ibidem, 06 and 28.09.2022, *El Mokni s.n.* (Herb. El Mokni!), ibidem, 09 September 2023, *El Mokni s.n.* (Herb. El Mokni!), ibidem, 30.09.2024, *El Mokni s.n.* (Herb. El Mokni!), ibidem, 19.09.2024, *El Mokni s.n.* (Herb. El Mokni, RO).

**C.1.2. *Periploca*** L., Sp. Pl.: 211 (1753).

***Periploca angustifolia*** Labill., in Icon. Pl. Syr. 2: 13 (1791).

Lectotype [designated by Browicz [75] (pag. 44 [first step] and Ferrer-Gallego [76] (pag. 29 [second step]). Oriente, s.d., Labillardière s.n. (G00177315! image available at https://www.ville-ge.ch/musinfo/bd/cjb/chg/adetail.php?id=180230&base=img&lang=en (accessed on 17 January 2025); isolectotypes, BM001014113!, FI055611!, FI055612!).

**Description.** *Periploca angustifolia* is an erect shrub or scrambler. A detailed morphological description was given by Venter [77] (pag. 126).

**Phenology in Tunisia.** Flowering and fruiting specimens have been seen throughout the year for many years.

**Global distribution and occurrence in Tunisia.** Its distribution covers the North African Sahara to Egypt; Malta, Spain, and Italy; around Sicily and Crete; Syria; and various other arid habitats [48,77]. In Tunisia, the plant has a very wide and extensive range of distribution from south to north, excluding acidic soils within the Numidian Flyschs (Kroumiria and some surroundings). More recently, the species was reported by the first author from many localities within the Monastir governorate (centraleastern part). The species is native to the country and is subject to overgrazing.

**Notes.** *Periploca angustifolia* can be morphologically confused with *P. laevigata* Ait. Besides their different distribution range, the two species can mainly be distinguished based on the following differences: the latter has leaves that are always opposite, 25–70 mm long, and follicles that are ca. 70–135 mm long, whereas the former has fasciculate leaves on older stems, usually 15–30(–35) mm long, and follicles that are 50–70 mm long [77] (pag. 125).

**Specimens examined.** TUNISIA. Bizerta: Tinja (Djebel Ichkeul), 37°08′37″ N, 009°40′11″ E, 10 m a.s.l., cracks in limestone/calcareous rocks, 28.04.2004, *El Mokni s.n.* (Herb. Univ. Bizerta); Zaghouan: Zaghouan-city (Djebel Zaghouan), 36°22′02″ N, 010°07′22″ E, 810 m a.s.l., cracks in limestone/calcareous rocks, 01.05.2015, *El Mokni s.n.* (Herb. El Mokni!); Sousse: Enfidha (Takrouna), 036°09′09” N, 010°20′18” E, 61 m a.s.l., cracks in limestone/calcareous rocks of scrublands, 28.10.2018, *El Mokni s.n.* (Herb. El Mokni!), ibidem, 27.10.2019, *El Mokni s.n.* (Herb. El Mokni!); Monastir: Ksar Hellal, 35°39′17″ N, 010°53′48″ E, 23 m a.s.l., along railways, 10.10.2022, *El Mokni s.n.* (Herb. El Mokni!), ibidem, 13.10.2023, *El Mokni s.n.* (Herb. El Mokni, RO); Gafsa: Sened, 34°27′44” N, 009°15′44” E, 650 m a.s.l., saxicolous vegetation within the *Juniperus phoenicea* L. scrublands, 25.12.2024, *El Mokni s.n.* (Herb. El Mokni!). 

**D. Rauvolfioideae** Kostel., Allg. Med.-Pharm. Fl. 3: 1054 (1834).

In Tunisia, this subfamily only includes 3 tribes (Vinceae, Plumerieae, Carisseae) and 3 subtribes (Vincinae, Catharanthinae, Thevetiinae) with 4 genera (*Carissa*, *Cascabela*, *Catharanthus* and *Vinca*) and 6 species. A key to genera of the Subfamily Rauvolfioideae in Tunisia is presented:

1. Leaves alternate; shrub or small tree with yellow flowers
*Cascabela*
1. Leaves opposite or whorled; climbers, herbs, shrubs or trees; flowers either white or pink, or if yellow then plants climbing22. Plants with sharp, stiff spines at many nodes; shrubs or scrambling climbers
*Carissa*
2. Plants without spines at nodes33. Plants erect without stolons; corolla mostly pink or white, tube ± cylindric
*Catharanthus*
3. Plants sprawling with stolons; corolla purple-blue (white), tube funnel-shaped
*Vinca*


**D.1. Vinceae** Duby, Bot. Gall. 1: 324 (1828).

**D.1.1. Vincinae** M.E. Endress, Phytotaxa 159(3): 178 (2015).

**D.1.1.1. *Vinca*** L., Sp. Pl. 1: 109 (1853).

A key to known species of the genus *Vinca* L. in Tunisia is presented:

1. Margins of leaves and calyx lobes minutely hairy
*V. major*
1. Margin of leaves and calyx lobes glabrous22. Corolla tube 9–11 mm; corolla limb 25–30 mm across; calyx lobes 3–4(5) mm
*V. minor*
2. Corolla tube 12–18 mm; corolla limb 30–45 mm across; calyx lobes 5–14 mm
*V. difformis*


***Vinca difformis*** Pourr., Mém. Acad. Sci. Toulouse 3: 333 (1788) subsp. ***difformis***

Lectotype **(designated here)**. France, Languedoc-Roussillon, Narbonne, A Fontfroide, s.d., Pourret s.n. (P04220074! image available at https://mediaphoto.mnhn.fr/media/1441370124371cCGwd1QE7k3ziNIQ; accessed on 17 January 2025).

**Description.** *Vinca difformis* is a perennial, rhizomatous, stoloniferous herb; leaves (9–) 25–100 × 7–55 mm, opposite on both fertile and sterile stems, orbicular to lanceolate, deltoid or ovate, glabrous, without cilia on the margin, petiolate; petiole 2.0–10.5 mm; inflorescence unifloral, axillary; flowers ebracteate, pedicellate; pedicels 1448 mm; calyx green; tube 1.2–2.5 mm, lobes (3–)5–14(–18) × 0.7–1.8 mm, triangular with 2 basal teeth, glabrous, frequently with a tuft of hairs at the apex; corolla blue-violet, sometimes whitish to whitish-blue at the mouth of the tube; tube 11–21 mm; lobes 15–22(–30) mm. style 4.5–6.5 mm; follicles 13–40 × 3–5 mm, torulose, brown; seeds 9.0–9.5 × 2.8–3.5 mm, narrowly ellipsoid, with longitudinally striated surface, warty alveolate, brown. 

**Phenology in Tunisia.** Flowering and fruiting specimens have been seen from May to December for many years. 

**Global distribution and presence in Tunisia.** *Vinca difformis,* an evergreen, flowering subshrubby plant, is native to Western Europe, including the Iberian Peninsula, France, the Italian Peninsula, and Sardinia. It is also native to Morocco, Algeria, and Tunisia in North Africa [10,48]. It was reportedly introduced to Great Britain and Ireland [30]. In Tunisia, the species is known only within Kroumiria (Jendouba governorate, northwestern Tunisia), where the last records by the first author were reported in 2024. The species is to be considered as naturalized in the country.

**Typification of the name *Vinca difformis*.** Pourret [78] (pag. 333) validly described *Vinca difformis,* providing a short diagnosis (“VINCA difformis) foliis ovato-lanceolatis glabris; floribus terminalibus irregularibus, calyce inaequali, tubo longiore”), followed by the symbol of Jupiter (♃), which means that the plant is perennial. “A Fontfroide” was also reported and this refers to a French Cistercian monastery located in southwest of Narbonne (Languedoc-Roussillon, south France).

We traced one specimen (barcode P04220074) at P (where Pourret’s collection and type are preserved; see [79]) bearing two branches of a plant and an original label (on the bottom-left corner of the sheet), reporting the *locus classicus* (“à Fontfroide”). Based on the label occurring on the bottom-right corner of the sheet, this exsiccatum is part of the “Collection de l’Abbé POURRET” and it can be considered part of the original material used by Pourret [78] (pag. 333) to describe *Vinca difformis*. The specimen matches the protologue and corresponds to the current concept of *Vinca difformis* s.l.; currently, two subspecies are recognized under *V. difformis*, i.e., subsp. *difformis* and subsp. *sardoa* Stearn (endemic to Sardinia, Italy). They differ from each other in terms of the leaves (larger and glabrous at the margins in subsp. *difformis* vs. smaller and minutely pubescent at the margins), calyx lobes (glabrous vs. ciliolate), and corolla (3.0–4.5 cm in diameter vs. 5–7 cm) (see, e.g., [80]).

**Specimens examined.** TUNISIA. Jendouba: Béni Métir, 36°44′22″ N, 08°44′17″ E, 475 m a.s.l., *Quercus* forests, 30.03.2006, *El Mokni s.n.* (Herb. Univ. Bizerta); ibidem, Oued Zéen, 36°49′27″ N, 008°50′00″ E, 355 m a.s.l., *Quercus* forests, 19.03.2018, *El Mokni s.n.* (Herb. Univ. Monastir, RO). 

***Vinca major*** L., Sp. Pl.: 209 (1753).

Lectotype [designated by Stearn [81] (pag. 79)]. Herb. Linn no. 299.3 (LINN! Image available at https://linnean-online.org/3044; accessed on 17 January 2025).

**Description.** *Vinca major* is a perennial, rhizomatous, stoloniferous herb; leaves 21–107 × 17–76 mm, opposite on both fertile and sterile stems, almost orbicular at the base of the stems to lanceolate at the apex, with ciliate margin, with 0.42–0.83 mm cilia, hairy midrib, petiolate; petiole 8–16 mm; inflorescence unifloral, axillary; flowers ebracteate, pedicellate; pedicels 16–48 mm; calyx green; tube 1.8–3.2 mm; lobes (8–)10.0–15.5 × (0.8–)1.0–1.5 mm, long triangular, with two basal teeth, with marginal cilia of 0.45–1.1 mm and an apical tuft of hairs; corolla blue-violet, sometimes whitish at the mouth of the tube; tube 15.5–20 mm; lobes 11–26 mm; style 4–5 mm; follicles 17–56 × 3–8 mm, torulose, brown; seeds 8.7–9.0 × 2.4–2.8 mm, narrowly ellipsoid, with striate-reticulate surface, brown.

**Phenology in Tunisia.** Flowering and fruiting specimens have been seen from June to November for many years.

**Global distribution and presence in Tunisia.** The plant is considered native in south Europe, from France to the west Balkans, and from Turkey eastwards to the Levant; additionally, it is an introduced/naturalized or casual alien in adjacent areas from the Atlantic coast (Ireland, Norway, Portugal, Spain), Belgium, Germany, and is also found in the Canary Islands, south central and southeast China, and the Americas [29]. For inland North Africa, it was only reported as cultivated for Morocco and Algeria (doubtfully escaping) and was naturalized for Tunisia [48]. After it was first reported [10], the taxon was reported many times by the first author within Kroumiria (Jendouba governorate, northwestern Tunisia), being last recorded in 2024. The species is to be considered as naturalized in the country

**Specimens examined.** TUNISIA. Jendouba: Aîn Draham, 36°46′34″ N, 008°40′54″ E, 715 m a.s.l., *Quercus spp.* forests, 4.04.2012, *El Mokni s.n.* (herb. Univ. Bizerta); ibidem, Fernana, 36°39′54″ N, 008°41′10″ E, 290 m a.s.l., *Quercus suber* L. forests, 18.03.2018, *El Mokni s.n.* (herb. Univ. Monastir, RO); ibidem, Tabarka, 36°57′30″ N, 008°45′17″ E, 9 m a.s.l., coastal vegetation, 27.02.2013, *El Mokni s.n.* (herb. Univ. Bizerta); ibidem, Tabarka, 36°55′40″ N, 008°47′28″ E, 30 m a.s.l., *Quercus suber* L. vegetation, 7.03.2017, *El Mokni s.n.* (herb. Univ. Monastir). 

***Vinca minor*** L., Sp. Pl.: 209 (1753).

Lectotype [designated by Stearn [81] (pag. 47)]. Herb. Linn no. 299.1 (LINN! image available at https://linnean-online.org/3042, accessed on 26 July 2024).

**Description.** *Vinca minor* is a perennial, rhizomatous, stoloniferous herb; leaves 15–52 × 7–25 mm, opposite on fertile stems, triverticillate or usually tetraverticillate at the apex of sterile stems, ovate, rarely lanceolate, glabrous, without cilia on the margin, petiolate; petiole 2–4(–8) mm. Inflorescence unifloral, axillary; flowers ebracteate, pedicellate; pedicels 7–38 mm; calyx green; tube 1.3–1.6 mm; lobes 3.5–4.3 × 0.8–1.6 mm, broadly triangular, with 2 basal teeth, glabrous; corolla blue-violet, sometimes whitish at the mouth of the tube; tube 9–14 mm; lobes 6–15 mm; style c. 4.5 mm; follicles 20–25 × 6–10 mm, somewhat torulose, brown; seeds 5.7–7.0 × 1.6–2.5 mm, narrowly ellipsoid, with their surface striated-reticulated, brown. 

**Phenology in Tunisia.** Flowering and fruiting specimens have been seen from May to December for many years.

**Global distribution and presence in Tunisia.** It is native to Europe, from Spain to the Caucasus [26] (pag. 163). It is reported as introduced in Portugal, Turkey, New Zealand, Central European Russia, China Southeast, Denmark, Great Britain, Ireland, West Siberia, and North America [30]. In continental Africa, the plant is only known to be found in Tunisia [82]. Since it was first reported [10], the plant was reported by the first author many times within Kroumiria (Jendouba governorate, northwestern of Tunisia), with a last report from Fernana (Jendouba governorate, northwestern of Tunisia) in 2024. The species can be considered as naturalized in the country.

**Specimens examined.** TUNISIA. Jendouba: Aîn Draham, Col des Ruines, 36°46′58″ N, 008°41′11″ E, 725 m a.s.l., *Quercus spp.* forests, 4.04.2012, *El Mokni s.n.* (Herb. Univ. Bizerta, RO).

**D.1.2. Catharanthinae** Pichon ex Boiteau in A. Aubrév. and J.-F. Leroy, Fl. Nouv.-Calédonie and Dépend. 10: 8 (1981).

**D.1.2.1. *Catharanthus*** G. Don, Gen. Hist. 4(1): 95 (1837).

***Catharanthus roseus*** (L.) G.Don, in Gen. Hist. 4: 95 (1837) ≡ *Vinca rosea* L., Syst. Nat. ed. 10,2: 944 (1759) ≡ *Lochnera rosea* (L.) Spach., Hist. Nat. Veg. Phan. 8: 526 (1839).

Lectotype [designated by Codd in Dyer et al. [83] (pag. 268)]. [Icon] Vinca, foliis oblongo-ovatis integerrimis, tubo floris longissimo caule ramoso fruticoso in Miller (1757: t. 186, image available at https://bibdigital.rjb.csic.es/viewer/13322/?offset=#page=62&viewer=picture&o=bookmark&n=0&q=; accessed on 17 January 2025).

**Description.** *Catharanthus roseus* is a subshrub or perennial herb up to 1 m tall, erect or decumbent; young stems puberulent; leaves obovate or elliptic, 2.5–9.0 × 1.0–3.5 cm, herbaceous, apex minutely apiculate; lateral veins 7–11 pairs; inflorescence ebracteate, pedicels green, 0.1–0.2 mm long, laxly puberulous to glabrous; flowers (3.0–)4.0–5.0(–5.6) cm long; sepals green, slightly connate at the base, sometimes slightly unequal, 2.7–4.7 × as long as wide, (2.0–)3.0–5.0 × 1.0–1.5 mm, outside laxly puberulous or glabrous, inside glabrous, sometimes towards the apex with some minute white hairs, entire, erect; corolla red to pink or white and then mostly with a pink or less often yellow eye; tube 2.5–3.0 cm, pilose inside, throat villous; lobes broadly obovate, 1.2–2.0 cm; follicles cylindrical, striate, 2.0–3.8 cm × ca. 3 mm, inside glabrous, dehiscent at the adaxial side; seeds numerous, grooved at one side, 1.0–2.0 × 0.5–0.8 mm. 

**Phenology in Tunisia.** Flowering and fruiting specimens have been seen throughout the year for many years.

**Iconography.** Plaizier [84] (pag. 4, Figure 1).

**Global distribution and presence in Tunisia.** Indigenous to Madagascar, the plant is cultivated and naturalized all over the world in the tropics and even sometimes in the subtropics [84]. In Tunisia, after its first report (Nabeul governorate, El Mokni 2018), the plant was reported in 2020 in Monastir-city (Monastir governorate, central eastern Tunisia) and was more recently reported in 2024 in Ksar-Hellal (Monastir governorate, central eastern Tunisia). The species can be considered as naturalized in the country.

**Specimens examined.** TUNISIA. Nabeul: Nabeul-city, 36°27′32″ N, 010°44′49″ E, 13 m a.s.l., roadsides, 11.12.2016, *El Mokni s.n.* (herb. Univ. Monastir, RO). Monastir, Ksar Hellal, 35°38′48″ N, 010°53′16″ E, 28 m a.s.l., ruderal habitats, 14.10.2024, *El Mokni s.n.* (Herb. El Mokni!).

**D.2. Plumerieae** E. Mey., Comm. Pl. Afr. Austr. 2: 188 (1838).

**D.2.1. Thevetiinae** A. DC. in DC. and A. DC., Prodr. 8: 342 (1844).

**D.2.1.1. *Cascabela*** Raf., in Sylva Tellur.: 162 (1838).

***Cascabela thevetia*** (L.) Lippold, Feddes Repert. 91: 52 (1980) ≡ *Cerbera thevetia* L., Sp. Pl.: 209 (1753).

Lectotype [designated by Lippold [85] (pag. 52)]. [Icon] Cerbera foliis linearibus, longissimus in Plumier’s Codex Boerhaavianus (image available in Burman 1755: Tab. XVII, https://www.biodiversitylibrary.org/item/15474#page/36/mode/1up; accessed on 17 January 2025).

**Description** (Figure 3). *Cascabela thevetia* is a vigorous tree up to 10 m tall with glabrous young branches; leaves petiolate, petioles 1–5 mm long, glabrous; lamina 70–170 × 5–14 mm, lanceolate to linear-elliptic, base and apex acute, firmly membranaceous, glabrous on both sides, secondary venation inconspicuous; inflorescences 8–10 cm long, 5–8-flowered; peduncles (5–)10–35(–50) mm long, glabrous; bracts 1.8–4.0 mm long, ovate–lanceolate, foliaceous, glabrous on both surfaces, with colleters on the adaxial base; pedicels 25–30 mm long, glabrous; sepals 5–13 × 2–4 mm, ovate–lanceolate, foliaceous, glabrous on both surfaces, with (0–)4–10 colleters in one row; corolla 45–65 mm long, yellow to orange; lower tube 12–20 × 2–5 mm, with internal retrorse hairs, upper tube 8–15 × 12–15 mm, glabrous, limb lobes 25–35 × 17–25 mm, obliquely oblong to oblong– ovate, erect, glabrous on both surfaces; anthers 2–3 mm long; pistil 14–18 mm long, glabrous; ovary 4.0 × 3.0 mm, glabrous; style 1.0–1.2 cm long, style head 2.0–3.0 × 2.5–3.0 mm long; drupe 25–35 × 20–45 mm, subglobose, glabrous, black, endocarp stony, irregularly deltoid, not lenticellate; seeds oblongate, 10–12 × 10 mm, white to yellowish. More details are available, e.g., in [86] (pag. 320) and [87] (pag. 363–364).

**Phenology in Tunisia.** Flowering and fruiting specimens have been seen from May to November/December for many years.

**Chromosome number.** 2n = 2x = 20 (sub *Thevetia peruviana* (Pers.) K.Schum [88,89]).

**Global distribution and occurrence in Tunisia.***Cascabela thevetia* is native to tropical America, from Mexico to Peru. The plant has been introduced and can be found, naturalized, in North America, the West Indies, Southern Asia, and Australia, and on many islands in the Pacific and Indian Ocean [30,90]. In Europe, *Cascabela thevetia* is reported as introduced to Cyprus, Spain, and the Canary Islands [90]. In continental Africa, the plant (sub *Thevetia peruviana*) was reported as introduced to many countries, including Angola, Chad, Guinea, and Senegal [90]; was reported as naturalized in Botswana, Burundi, Ethiopia, Rwanda, and Zimbabwe; and was reported as invasive in Kenya, Malawi, South Africa, Tanzania, Uganda, and Zambia [91]. In inland North Africa, there was no indication about any report of the species until now [30]; thus, our report from Tunisia here is the first to identify it as casual alien.

*Cascabela thevetia* was introduced and cultivated as ornamental in Tunisia, and was here reported for the first time from the following two localities (spontaneous plants and seedlings were found not far from the cultivated individuals and, as a consequence, the species can be considered as casual in the country):

> It was first recorded in central eastern Tunisia (Monastir region, Monastir-city, 22.08.2019), where several shrubby individuals were found near high-planted trees during the blooming–fruiting period within a limited border area not far away the central market of Monastir-city in an area of about one hectare. Seedlings were reported within the same locality on 18 December 2024.
> It was recorded a second time from the northeastern Tunisia (Tunis region, Bab Alouia, 6 July 2021), where we found 3 flowering–fruiting shrubby individuals in the border of an area planted with *Yucca* spp. within a ruderal habitat.

**Taxonomic notes.** *Cascabela* Raf. and *Thevetia* Adans (= *Cerbera* L.) are two taxa mainly distinguished based on the contrasting flower and fruit structure and seed shape. *Cascabela* species show flowers with yellow infundibuliform corollas, suprastaminal finger-like appendages, black drupes with stony endocarps, and non-compressed seeds with reduced wings, whereas Thevetia species has flowers with yellow to whitish hypocrateriform or infundibuliform corollas, suprastaminal deltoid-like appendages, red drupaceous fruits with four fibrous endocarps, and compressed seeds with or without wings [86,87].

*Cascabela thevetia* may be confused with *C. thevetioides* (Kunth) Lippold (a Mexican endemic species), from which it can easily be distinguished by its corolla tube size, 12–17 mm (vs. 20–30 mm in *C. thevetioides*), its glabrous leaf indumentum (vs. tomentose in *C. thevetioides*), and its inconspicuous secondary veins (vs. exposed in *C. thevetioides*) [86].

**Notes on the type of *Cerbera thevetia*.** Lippold [85] (pag. 52) correctly lectotypified the Linnaean name using Plumier’s illustration of Cerbera foliis linearibus, longissimus in “Codex Boerhaavianuns”. Linnaeus [52] (pag. 209) reported, after the short diagnosis (“Cerbera foliis linearis longissimus confertis”), the synonym “Ahouaj nerii folio, flore luteo. Plum. Spec. 20” which referred, in turn, to the page 20 of Plumier’s *Catalogus plantarum Americanarum*, a supplement to his *Nova Plantarum Americanarum Genera* [92], where no illustration was published. Plumier prepared a series of drawings during his trip to the West Indies (second half of the century XVII); these drawings are preserved at the library of the Muséum National d’Histoire Naturelle in Paris and copies of 508 of them were made in Paris in 1733 for Hermann Berhaave in Leiden and these are known as the “Codex Boerhaavianus”. After Boerhaave’s death, these drawings were acquired by Johan Burman, who published them in his *Plantarum Americanarum Fasciculum* [93] (see, e.g., [94] (pag. 323–324). Plate XVII is Plumiers’ Cerbera foliis linearibus, longissimus.

**Specimens examined.** TUNISIA. Monastir: Monastir-city, 35°46′21″ N, 010°49′59″ E, 11 m a.s.l., ruderal habitats, 22.08.2019, *El Mokni s.n.* (Herb. El Mokni!); ibidem, 27.06.2023, *El Mokni s.n.* (Herb. El Mokni, RO); Tunis: Tunis-city (Bab Alouia), 36°47′18″ N, 010°10′50″ E, 5 m a.s.l., within roadsides, 06.07.2021, *El Mokni s.n.* (Herb. El Mokni!).

**D.3. Carisseae** Dumort, Anal. Fam. Pl.: 26 (1829). 

**D.3.1.1. *Carissa*** L., Mant.: 52 (1767).

***Carissa macrocarpa*** (Eckl.) A.DC., Prod. 8: 336 (1844) ≡ *Arduina macrocarpa* Eckl., S. African J. 1: 372 (1830) ≡ *Jasminonerium macrocarpum* (Eckl.) Kuntze, Revis. Gen. Pl. 2: 414 (1891).

Neotype [designated by Leeuwenberg and Van Dilst [95] (pag. 27)]. Republic of South Africa, atal, between Umzinto and Durban, 23 November 1958, Werdermann and Oberdieck 1211 [B *non videmus fide* Leeuwenberg and Van Dilst 2001: 27; isoneotypes at A00071057 (image available at https://plants.jstor.org/stable/viewer/10.5555/al.ap.specimen.a00071057?loggedin=true; accessed on 17 January 2025), K (non videmus fide Leeuwenberg and Van Dilst 2001: 27), US00731165! (http://n2t.net/ark:/65665/m397fb20d2-1468-4e98-9f15-4216c0e53fe3; accessed on 17 January 2025), WAG (*non videmus fide* Leeuwenberg and Van Dilst 2001: 27)].

**Figure 4:**
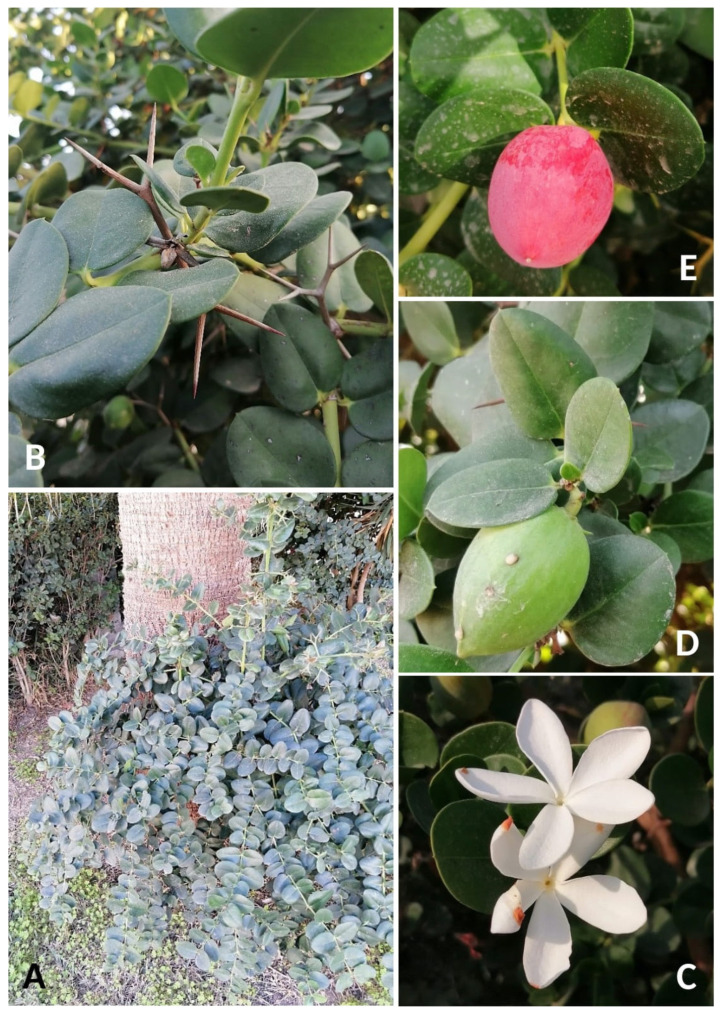
*Carissa macrocarpa* in Tunisia. (**A**) Habit; (**B**) bifurcate robust spines; (**C**) corolla 5-lobed overlapping to left; (**D**) immature ovoid fruit; (**E**) mature fruit (photographs by R. El Mokni).

**Description** (Figure 4). *Carissa macrocarpa* is a vigorous, spreading woody shrub with abundant, gummy white sap that may reach a height of 4.5–5.5 m and an equal breadth; branches are armed with stout, double-pronged thorns of up to 5 cm long; leaves are evergreen, opposite, broad–ovate, 2.5–5 cm long, dark green, glossy, and leathery; flowers are sweetly fragrant, white, and 5-lobed with tubes to 5 cm and are borne singly or with several together at the tips of branches; fruits are ovoid to spheroid, standing at 6.25 cm long and 4 cm wide; upon ripening, the tender, smooth skin turns a bright magenta-red; seeds are small, flat, and brown, and there are up to 16 per fruit. More details are available, e.g., in [95] (pag. 146), [96] (pag. 27–28, 30), [97] (pag. 2–3), and [98].

**Phenology in Tunisia.** Flowering and fruiting specimens have been seen from March to December for many years.

**Figure 5:**
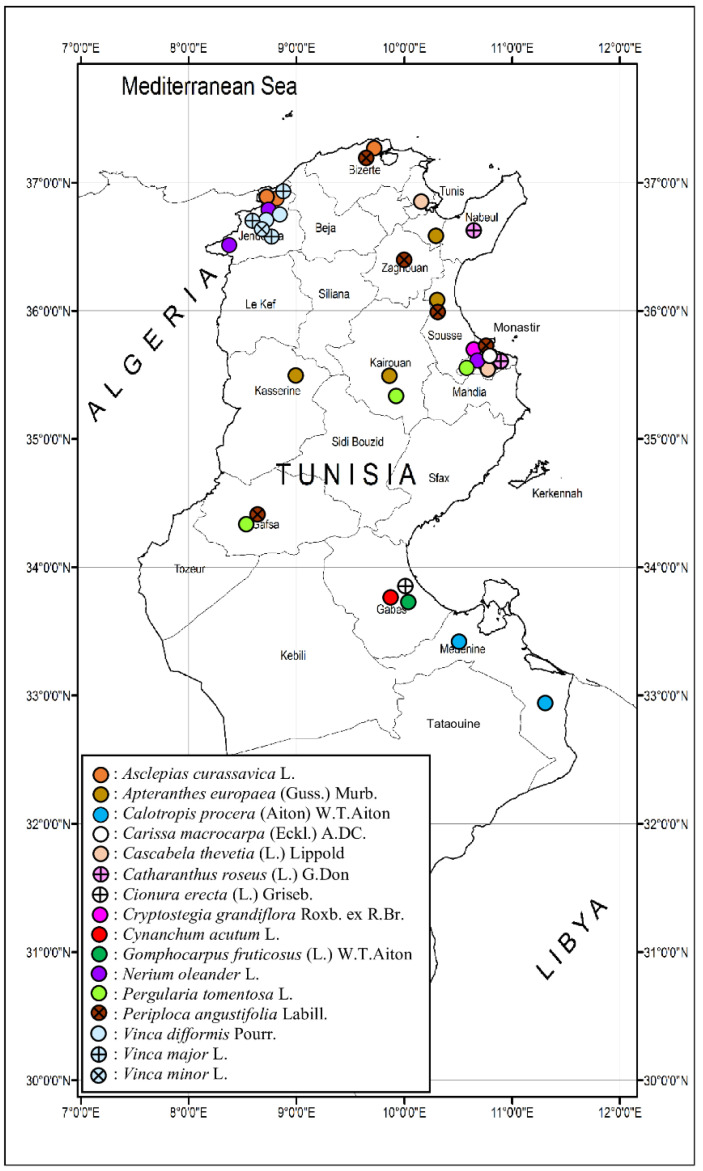
A map of all localities from where Tunisian Apocynaceous vouchers were sampled, including the new species to the flora of Tunisia found during the present study.

**Iconography.** Leeuwenberg and Van Dilst [95] (pag. 28, Figure 5)

**Chromosome number.** 2n = 2x = 66 [95] (pag. 637) and [99] (pag. 146).

**Global distribution and occurrence in Tunisia.** *Carissa macrocarpa* is native to central and southern Africa. It is now widespread as an ornamental throughout the Lucayan Archipelago, the Caribbean region, Central and North America, and parts of Asia ([98,100]. For North Africa, it seems no report of the taxon was listed yet in the Euro+Med Plant Base [48], in the *African Plant Database* [101], or in the *Plant of the Word online* [29]. Conversely, in the Global Biodiversity Information Facility [102], *Carissa macrocarpa* was reported as being present in the Canary Islands, Morocco, and Tunisia without any naturalization. Thus, this is its first report as a casual alien in Tunisia. 

*Carissa macrocarpa* was reported here for the first time in Tunisia from two localities (the species can be considered as casual in the country):

> It was first recorded in the wild in the central eastern part of Tunisia (Monastir region, Monastic-city, 18 May 2016), where about 6 shrubby blooming–fruiting individuals were found within a limited area not far away a huge individual in an area of about 100 m^2^.
> Its second recording in the wild was in the central eastern part of Tunisia (Monastir region, Marina, 31 December 2020), where three flowering–fruiting individuals were found at the base of the stipe of a planted Washingtonia robusta H. Wendl. in a ruderal habitat.

**Taxonomic notes.** *Carissa macrocarpa* can be distinguished easily from other closely related species by its corolla lobes overlapping to left, as long as or longer than tube. Related species (mainly *C. spinarum* L.) show corolla lobes overlapping to right with tepals shorter than the tube [96] (pag. 146). Moreover, *C. macrocarpa* shows corolla tube (9-)10.5–18.5 mm long with fruits 2–3.5 cm long, whereas in *C. bispinosa* (L.) Desf. ex Brenan, corolla tube 4.8–9 mm long and fruits ca. 1–2 cm long [95] (pag. 7).

**Specimens examined.** TUNISIA. Monastir: Monastir-city, 35°46′22″ N, 010°50′02″ E, 12 m a.s.l., ruderal habitats, 18.05.2016, *El Mokni s.n.* (Herb. El Mokni!), ibidem, 31.12.2020, *El Mokni s.n.* (Herb. El Mokni, RO).

## 4. Conclusions

All new additions reported (Figure 5) here are regarded as casual aliens, except for *Asclepias curassavica*, which seems to be naturalizing. Horticultural trade is considered the most likely pathway for their introduction. Dissemination over long distances (invasion) is promised mainly by the production of fruits (in the case of *Carissa* and *Cascabela*) and seeds bearing coma (*Asclepias* and *Cryptostegia*) along ruderal communities. Regarding the records reported here, the floristic account of the Apocynaceae family in Tunisia now involves 14 genera (including 16 species, with no Tunisian endemics among them), 7 subtribes, 8 tribes, and 4 subfamilies, where the richest is the Asclepiadoideae. There were new records of 3 subfamilies (except Apocynoideae), 4 tribes (Asclepiadeae, Carisseae, Cryptolepideae, Plumerieae of which three are here their first report), 2 subtribes (Asclepiadinae and Thevetiinae and the latter is here its first report), 4 genera (all of which were reported here at the national level for the first time, and 3 of which were found in inland North Africa and as examples of Mediterranean floristic diversity for the first time), and 4 species.

## Data Availability

Data are contained within the article.
